# Self-reported quality of life of adolescents with cerebral palsy: a cross-sectional and longitudinal analysis

**DOI:** 10.1016/S0140-6736(14)61229-0

**Published:** 2015-02-21

**Authors:** Allan Colver, Marion Rapp, Nora Eisemann, Virginie Ehlinger, Ute Thyen, Heather O Dickinson, Jackie Parkes, Kathryn Parkinson, Malin Nystrand, Jérôme Fauconnier, Marco Marcelli, Susan I Michelsen, Catherine Arnaud

**Affiliations:** a Institute of Health and Society, Newcastle University, Royal Victoria Infirmary, Newcastle upon Tyne, UK; b Klinik für Kinder und Jugendmedizin, Universität Lübeck, Lübeck, Germany; c Institute of Cancer Epidemiology, Universität Lübeck, Lübeck, Germany; d INSERM, UMR 1027, Paul Sabatier University, Toulouse, France; e School of Nursing and Midwifery, Queen's University Belfast, Medical Biology Centre, UK; f Göteborg University, The Queen Silvia Children's Hospital, Sweden; g UJF-Grenoble 1/CNRS/CHU de Grenoble/TIMC-IMAG UMR 5525/Themas, France; h AUSL Viterbo, Italy; i National Institute of Public Health, Denmark; j Purpan, Clinical Epidemiology Unit, Toulouse, France

## Abstract

**Background:**

Children with cerebral palsy who can self-report have similar quality of life (QoL) to their able-bodied peers. Is this similarity also found in adolescence? We examined how self-reported QoL of adolescents with cerebral palsy varies with impairment and compares with the general population, and how factors in childhood predict adolescent QoL.

**Methods:**

We report QoL outcomes in a longitudinal follow-up and cross-sectional analysis of individuals included in the SPARCLE1 (childhood) and SPARCLE2 (adolescent) studies. In 2004 (SPARCLE1), a cohort of 818 children aged 8–12 years were randomly selected from population-based cerebral palsy registers in nine European regions. We gathered data from 500 participants about QoL with KIDSCREEN (ten domains); frequency of pain; child psychological problems (Strengths and Difficulties Questionnaire); and parenting stress (Parenting Stress Index). At follow-up in 2009 (SPARCLE2), 355 (71%) adolescents aged 13–17 years remained in the study and self-reported QoL (longitudinal sample). 76 additional adolescents self-reported QoL in 2009, providing data for 431 adolescents in the cross-sectional sample. Researchers gathered data at home visits. We compared QoL against matched controls in the general population. We used multivariable regression to relate QoL of adolescents with cerebral palsy to impairments (cross-sectional analysis) and to childhood QoL, pain, psychological problems, and parenting stress (longitudinal analysis).

**Findings:**

Severity of impairment was significantly associated (p<0·01) with reduced adolescent QoL on only three domains (Moods and emotions, Autonomy, and Social support and peers); average differences in QoL between the least and most able groups were generally less than 0·5 SD. Adolescents with cerebral palsy had significantly lower QoL than did those in the general population in only one domain (Social support and peers; mean difference −2·7 [0·25 SD], 95% CI −4·3 to −1·4). Pain in childhood or adolescence was strongly associated with low adolescent QoL on eight domains. Childhood QoL was a consistent predictor of adolescent QoL. Child psychological problems and parenting stress in childhood or their worsening between childhood and adolescence predicted only small reductions in adolescent QoL.

**Interpretation:**

Individual and societal attitudes should be affected by the similarity of the QoL of adolescents with and without cerebral palsy. Adolescents with cerebral palsy need particular help to maintain and develop peer relationships. Interventions in childhood to alleviate psychological difficulties, parenting stress, and especially pain, are justified for their intrinsic value and for their longer term effect on adolescent QoL.

**Funding:**

SPARCLE1 was funded by the European Union Research Framework 5 Program (grant number QLG5-CT-2002-00636), the German Ministry of Health GRR-58640-2/14, and the German Foundation for the Disabled Child. SPARCLE2 was funded by: Wellcome Trust WT086315 A1A (UK and Ireland); Medical Faculty of the University of Lübeck E40-2009 and E26-2010 (Germany); CNSA, INSERM, MiRe–DREES, and IRESP (France); Ludvig and Sara Elsass Foundation, The Spastics Society and Vanforefonden (Denmark); Cooperativa Sociale “Gli Anni in Tasca” and Fondazione Carivit, Viterbo (Italy); Göteborg University—Riksforbundet for Rorelsehindrade Barn och Ungdomar and the Folke Bernadotte Foundation (Sweden).

## Introduction

WHO defines quality of life (QoL) as “the individual's perception of their position in life in the context of the culture and value system in which they live, and in relation to their goals, expectations, standards, and concerns”.^1^ Thus, QoL is subjective and should be self-reported whenever possible. Promotion of a good QoL is important for all but might be neglected in young people with disabilities due to an emphasis on trying to remedy their impairments. Young people with cerebral palsy are often studied as exemplars of children with disabilities because its severity, patterns of motor involvement, and associated impairments, such as communication, intellectual ability, and epilepsy, vary widely and persist across the life course. Prevalence of cerebral palsy has remained stable for the past 40 years at 2–3 per 1000 livebirths,^2^ in countries where accurate data are available.

The European study SPARCLE showed that the QoL of children aged 8–12 years with cerebral palsy who could self-report was similar to that of children in the general population; it showed little variation with impairment on most domains, but pain was common and associated with low QoL on all domains.^3^ A study in the USA reported similar findings.^4^ QoL of adolescents with cerebral palsy has been studied^5^, ^6^, ^7^, ^8^, ^9^, ^10^, ^11^ but often with small sample sizes or an inappropriate method to capture QoL.

The SPARCLE study has also assessed QoL at age 13–17 years in the same young people who were visited at age 8–12 years. We report a cross-sectional analysis of the SPARCLE cohort that investigates how QoL of adolescents with cerebral palsy compares with that of adolescents in the general population and how it varies with type and severity of impairment. We also report a longitudinal analysis in which we assess childhood factors that are amenable to intervention and known to be associated with lower QoL in childhood:^3^, ^12^ pain, psychological problems, and parenting stress. Our longitudinal analysis aims to assess whether QoL changes between childhood and adolescence, and whether QoL in adolescence is predicted by QoL in childhood, pain in childhood and adolescence, psychological problems and parenting stress in childhood, and their changes between childhood and adolescence.

## Methods

### Study design and participants

**Figure 1:**
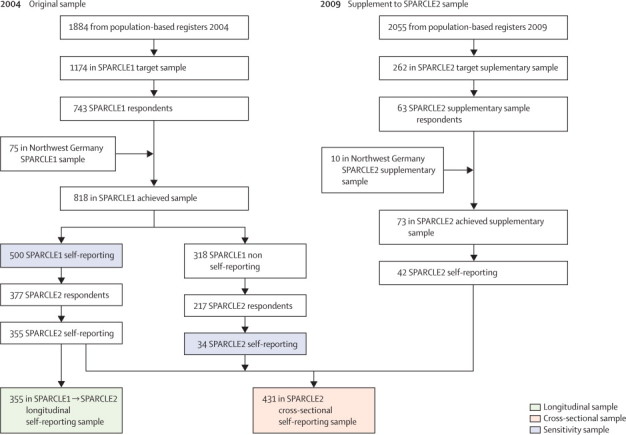
Study profile

The methods of the SPARCLE study have been described in detail elsewhere^13^, ^14^, ^15^, ^16^ and we summarise them here. We randomly sampled children born between July 31, 1991, and April 1, 1997 from population-based registers of children with cerebral palsy in eight European regions (Table 1, Table 2) that share a standardised definition of cerebral palsy.^17^ 743 (63%) of 1174 target families identified from registers joined the study. We also included one other region (northwest Germany) that had 75 children with cerebral palsy ascertained from many sources (figure 1).

**Table 1:** Sociodemographic characteristics of young people with cerebral palsy who self-reported quality of life (QoL)

		**Cross-sectional sample (adolescence only) (n=431)**	**Longitudinal sample (n=355)**
**Region of residence**			
East Denmark		54 (13%)	48 (14%)
France			
	Southeast France	43 (10%)	31 (9%)
	Southwest France	38 (9%)	33 (9%)
Southwest Ireland		50 (12%)	47 (13%)
Central Italy		17 (4%)	16 (5%)
West Sweden		40 (9%)	36 (10%)
UK			
	North England	73 (17%)	52 (15%)
	Northern Ireland	65 (15%)	53 (15%)
	Northwest Germany	51 (12%)	39 (11%)
**Sex**			
Male		251 (58%)	202 (57%)
Female		180 (42%)	153 (43%)
**Age, years (when interviewed in adolescence)**			
<13		27 (6%)	21 (6%)
13		100 (23%)	80 (23%)
14		86 (20%)	74 (21%)
15		91 (21%)	74 (21%)
16		76 (18%)	64 (18%)
17		47 (11%)	39 (11%)
>17		4 (1%)	3 (1%)

Data are n (%). *Cross-sectional analysis did not use these predictors.

**Table 2:** Impairments and predictors of quality of life (QoL) for young people with cerebral palsy who self-reported QoL

		**Cross-sectional sample (adolescence only) (n=431)**	**Longitudinal sample (n=355)**	
			Childhood	Adolescence
**Impairments**				
Gross Motor Function Classification System				
	I Walks and climbs stairs, without limitation	199 (46%)	145 (41%)	172 (49%)
	II Walks with limitations	83 (19%)	93 (26%)	67 (19%)
	III Walks with assistive devices	58 (13%)	61 (17%)	46 (13%)
	IV Unable to walk, limited self-mobility	52 (12%)	39 (11%)	37 (10%)
	V Unable to walk, severely limited self-mobility	38 (9%)	17 (5%)	33 (9%)
	Missing	1 (<1%)	0	0
Bimanual Fine Motor Function				
	I Without limitation	201 (47%)	164 (46%)	170 (48%)
	II & III Moderate restrictions	187 (43%)	161 (45%)	151 (43%)
	IV & V Severe restrictions	40 (9%)	30 (8%)	33 (9%)
	Missing	3 (1%)	0	1 (<1%)
Seizures in the previous year				
	No seizures (either with or without medication)	393 (91%)	328 (92%)	326 (92%)
	Seizures	33 (8%)	27 (8%)	27 (8%)
	Missing	5 (1%)	0	2 (1%)
Feeding				
	Feeds by mouth with no problems	394 (91%)	317 (89%)	327 (92%)
	Feeds by mouth with difficulty, or by tube	35 (8%)	38 (11%)	26 (7%)
	Missing	2 (<1%)	0	2 (1%)
Communication				
	Normal	352 (82%)	289 (81%)	298 (84%)
	Communication difficulties	77 (18%)	66 (19%)	55 (15%)
	Missing	2 (<1%)	0	2 (1%)
Intellectual impairment				
	IQ >70	285 (66%)	258 (73%)	248 (70%)
	IQ ≤70	145 (34%)	94 (26%)	107 (30%)
	Missing	1 (<1%)	3 (1%)	0
Cerebral palsy subtype				
	Unilateral spastic	175 (41%)	152 (43%)	152 (43%)
	Bilateral spastic	210 (49%)	169 (48%)	171 (48%)
	Dyskinetic	29 (7%)	25 (7%)	22 (6%)
	Ataxic	12 (3%)	8 (2%)	8 (2%)
	Missing	5 (1%)	1 (<1%)	2 (1%)
Postulated predictors of QoL*				
Self-reported frequency of pain in previous week				
	None	..	164 (46%)	109 (31%)
	Once or twice	..	97 (27%)	121 (34%)
	Frequent	..	87 (25%)	122 (34%)
	Missing	..	7 (2%)	3 (1%)
Total difficulties score of Strengths and Difficulties Questionnaire (parent reported)				
	Normal (<14)	..	231 (65%)	229 (65%)
	Borderline (14–16)	..	50 (14%)	57 (16%)
	Abnormal (>16)	..	74 (21%)	67 (19%)
	Missing	..	0	2 (1%)
Total stress score of Parenting Stress Index				
	Normal (<86)	..	242 (68%)	226 (64%)
	Borderline (86–90)	..	29 (8%)	23 (6%)
	Abnormal (>90)	..	82 (23%)	99 (28%)
	Missing	..	2 (1%)	7 (2%)

Data are n (%).

* Cross-sectional analysis did not use these predictors.

The 818 children who entered the study were interviewed in 2004–05, aged 8–12 years (SPARCLE1), and followed up in 2009–10, aged 13–17 years (SPARCLE2) when 594 (73%) joined the study. Predictors of those that dropped out or declined to participate have been reported.^14^, ^16^ The 355 adolescents who were able to self-report their QoL in both SPARCLE1 and SPARCLE2 constitute the longitudinal sample (figure 1). The cross-sectional sample was composed of these 355 adolescents and an additional 34 adolescents who had self-reported their QoL in SPARCLE2 but not in SPARCLE1 and 42 adolescents who had not participated in but were eligible for SPARCLE1. We added this supplementary sample to maintain statistical power for cross-sectional analyses and follow-up to adulthood. We obtained ethical approval, or a statement that only registration was required, as appropriate to each country. We obtained signed consent from all parents and from young people.

### Procedures

Research associates, who had met in Newcastle for joint training in the study procedures, visited families in their homes to administer questionnaires to young people and their parents using the same questionnaires in childhood and adolescence. Young people reported their QoL with KIDSCREEN, a European questionnaire with strong psychometric properties, designed for children and adolescents.^11^, ^18^, ^19^ It has 52 items that ask about QoL in the previous week across ten domains (table 3). Items are scored on a 5-point scale and, within each domain, item scores are transformed to Rasch person parameters with an algorithm that gives young people in the reference population a mean score of 50 with an SD of 10, higher scores show better QoL.^18^ One item on the Physical wellbeing domain was amended from “able to run well” to “able to get about easily” to make it more suitable for young people with cerebral palsy. Young people reported their frequency of pain during the previous week, which we classified as no pain, one or two episodes of pain, or frequent pain.

**Table 3:** Description of each KIDSCREEN domain

	**Number of items**	**Perceptions assessed**
Physical wellbeing	5	Physical activity, energy, and fitness
Psychological wellbeing	6	Positive emotions and satisfaction with life
Moods and emotions	7	Negative moods, boredom, and stress
Self-perception	5	Satisfaction with self, body appearance, and body image
Autonomy	5	Freedom of choice and self-determination in leisure time
Relationships with parents	6	Interactions and relationships with parents and the socioemotional atmosphere at home
Social support and peers	6	Social support available from friends and peers
School life	6	Learning and feelings about school and teachers
Financial resources	3	Adequacy of pocket money relative to peers
Social acceptance	3	Social acceptance or rejection by peers, including bullying

Parents provided information about their child's impairments (walking ability as captured by the Gross Motor Function Classification System [GMFCS],^20^ fine motor function,^21^ seizures, feeding, communication, and intellectual ability^22^), family structure, and parents' educational qualifications. The type of cerebral palsy was available from the registers. Parents also completed the Strengths and Difficulties Questionnaire (SDQ); we used the total difficulties score (range 0–40; clinical problem >16; SD in general population 7) as a measure of the young person's psychological problems.^23^ Parents completed the SDQ to ensure consistency between childhood and adolescence. We assessed parenting stress at both timepoints with the total stress score of the Parenting Stress Index Short Form (PSI) (range 40–140; clinical stress >90; SD in general population 15).^24^

KIDSCREEN self-reported data from the general population were available from the developers of KIDSCREEN for 7539 adolescents of similar age in five countries in the SPARCLE study:^18^ France, Germany, Ireland, Sweden, and the UK. Similar data were also available for 933 adolescents aged 13–17 years in the general population in Denmark, collected during the course of SPARCLE2.

### Statistical methods

We report the statistical methods in full in the appendix. We did analyses separately for each of the ten KIDSCREEN domains. We first examined the psychometric properties of the self-reported KIDSCREEN scores in adolescents with cerebral palsy. We based subsequent analyses on ten imputed datasets with no missing values, generated by multiple imputation;^25^ we estimated 95% CIs by bootstrapping^26^ with 100 replications per domain for each of the ten imputed datasets. To compare adolescents with cerebral palsy and those in the general population, we selected two controls from the general population for each adolescent with cerebral palsy, matching for age, sex, and country; matching constraints decreased the sample with cerebral palsy to 399 (appendix). We estimated the mean difference of the QoL of adolescents with cerebral palsy and their matched controls. We then used multivariable linear regression, adjusted for age, sex, and region, to analyse the relation between adolescent QoL and impairment (cross-sectional analysis), and between adolescent QoL and childhood QoL (longitudinal analysis, baseline model). We developed four further longitudinal models by adding pain, SDQ, and PSI to the baseline model, both separately and in combination (appendix). Finally, we undertook sensitivity analyses around dropout for both the cross-sectional and longitudinal analyses. The criterion for statistical significance was that the 95% CIs did not include zero; we also inspected 99% CIs.

### Role of the funding source

The funders of the study had no role in study design, data collection, data analysis, data interpretation, or writing of the report. The corresponding author had full access to all the data in the study and had final responsibility for the decision to submit for publication.

## Results

Sociodemographic characteristics and impairments for the cross-sectional (n=431) and longitudinal (n=355) samples are shown in Table 1, Table 2. Children assessed in SPARCLE1 had a mean age of 10·4 years and adolescents assessed in SPARCLE2 15·1 years. On average, levels of psychological difficulties and parenting stress were similar in childhood and adolescence, but adolescents reported more frequent pain than did children. Spearman rank correlations [ρ] between gross motor function, bimanual fine motor function, feeding, IQ, and communication were significant, ranging from 0·2 to 0·5, p<0·0001; in childhood and adolescence, SDQ and PSI scores were highly correlated (0·6, p<0·0001); pain and SDQ scores were very weakly correlated (0·11, p=0·04); pain and PSI scores were not significantly correlated (0·09, p=0·08) (data not shown).

Table 4 shows the basic psychometric properties of KIDSCREEN in 431 adolescents with cerebral palsy included in the cross-sectional sample, which were previously reported in children with cerebral palsy.^3^ The Relationships with parents, Financial resources, and Social acceptance domains showed ceiling effects, with 19% (Relationships with parents), 27% (Financial resources), and 55% (Social acceptance) of adolescents having the maximum score. Cronbach's α values were between 0·70 and 0·90, as recommended.^27^

**Table 4:** KIDSCREEN scores on each domain for self-reporting adolescents with cerebral palsy (cross-sectional sample, n=431)

	**n (%)**	**Mean (SD)**	**Floor (%)**	**Ceiling (%)**	**Cronbach's α**
Physical wellbeing	427 (99%)	49·3 (10·6)	<1%	7%	0·76
Psychological wellbeing	429 (>99%)	48·4 (9·3)	<1%	7%	0·81
Moods and emotions	429 (>99%)	51·3 (10·0)	<1%	10%	0·83
Self-perception	427 (99%)	51·3 (10·0)	<1%	15%	0·70
Autonomy	428 (99%)	50·7 (9·7)	1%	14%	0·73
Relationships with parents	430 (>99%)	50·8 (9·6)	<1%	19%	0·80
Social support and peers	429 (>99%)	46·4 (12·5)	4%	7%	0·86
School life	425 (99%)	53·3 (10·2)	<1%	10%	0·83
Financial resources	419 (97%)	50·2 (9·9)	1%	27%	0·85
Social acceptance	424 (98%)	50·4 (10·2)	<1%	55%	0·76

In the reference population, each domain score has a mean of 50 with an SD of 10.^18^ Cronbach's α is a measure of the reliability of the scores.^27^ Floor=percentage of scores with the minimum values. Ceiling=percentage of scores with the maximum values.

**Figure 2:**
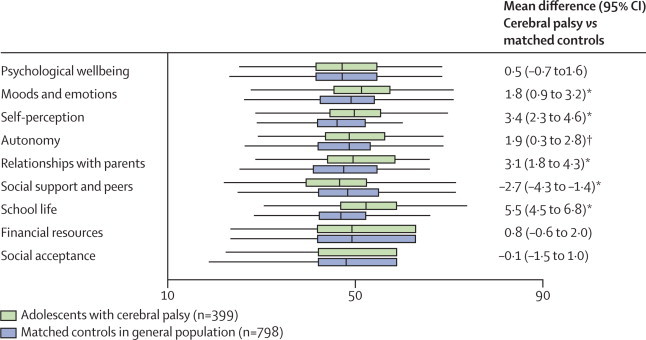
Box and whisker plots and mean differences of KIDSCREEN scores for adolescents with cerebral palsy and matched controls Boxes show median (IQR) and whiskers show adjacent values.^28^ For the Social acceptance and Financial resources domains, more than 25% of the values took the maximum value; therefore the upper adjacent values and, for Social acceptance of adolescents with cerebral palsy, the median, do not appear on the plots. The scales for each domain have mean 50 (SD 10) in the reference population. The Physical wellbeing domain was omitted from comparisons because one item had been amended to make it more suitable for young people with cerebral palsy. Statistical significance: *99% CI excluding zero; †95% CI excluding zero.

Compared with matched adolescents in the general population, adolescents with cerebral palsy had better QoL in five domains: Moods and emotions, Self-perception, Autonomy, Relationships with parents, and School life; conversely, they had worse QoL in the Social support domain (figure 2). Sensitivity analyses, which imputed missing KIDSCREEN scores for those who dropped out between childhood and adolescence, gave much the same results (data not shown).

We assessed variations in QoL by impairment type and severity for adolescents in the cross-sectional analysis. For five domains (Psychological wellbeing, Self-perception, Relationships with parents, School life, and Financial resources) QoL was not significantly associated with any type of impairment; for four domains (Moods and emotions, Autonomy, Social support and peers, and Social acceptance) it was significantly associated (99% CI excluding zero, for one or more type of impairment); and for the Physical wellbeing domain, the association was of marginal significance (95% CI excluding zero, for one or more type of impairment; table 5). Sensitivity analysis showed associations that were not as strong as those in table 5 and not significant for the Physical wellbeing and Social acceptance domains (data not shown).

**Table 5:** Linear regression models relating KIDSCREEN scores on each domain to type and level of impairment in adolescents with cerebral palsy (cross-sectional sample, n=431)

		**Physical wellbeing**	**Psychological wellbeing**	**Moods and emotions**	**Self-perception**	**Autonomy**	**Relationships with parents**	**Social support and peers**	**School life**	**Financial resources**	**Social acceptance**
Initial models considering each impairment separately*											
Gross Motor Function Classification System											
	I Walks and climbs stairs, without limitation	0·0 (reference)	0·0	0·0	0·0	0·0	0·0	0·0	0·0	0·0	0·0
	II Walks with limitations	−1·6 (−4·2 to 0·9)	−0·7 (−2·0 to 1·5)	−0·6 (−3·1 to 2·9)	−1·3 (−3·8 to 1·2)	−3·3 (−5·6 to −0·9)†	0·7 (−1·6 to 3·1)	−3·0 (−6·2 to 0·0)	0·0 (−2·4 to 2·3)	−0·9 (−3·6 to 1·9)	−1·0 (−3·6 to 1·5)
	III Walks with assistive devices	−2·5 (−5·5 to 0·6)	−0·5 (−3·6 to 2·5)	0·7 (−2·2 to 3·6)	0·3 (−2·6 to 3·3)	−3·9 (−6·6 to −1·0)†	1·1 (−1·9 to 4·1)	−2·7 (−6·6 to 1·0)	1·5 (−1·7 to 4·8)	−1·0 (−3·8 to 1·9)	1·0 (−2·0 to 3·9)
	IV Unable to walk, limited self-mobility	−1·5 (−4·8 to 2·1)	0·7 (−2·4 to 3·6)	−1·0 (−4·4 to 2·3)	−2·1 (−5·1 to 0·9)	−3·1 (−5·9 to −0·2)‡	1·2 (−1·7 to 4·1)	−4·9 (−8·7 to −1·3)†	0·0 (−3·8 to 4·0)	1·2 (−1·7 to 4·1)	−1·2 (−4·5 to 1·7)
	V Unable to walk, severely limited self-mobility	−3·0 (−7·1 to 1·0)	−1·2 (−4·5 to 2·2)	0·1 (−3·5 to 3·9)	−0·4 (−4·0 to 3·2)	−3·4 (−7·1 to 0·4)	1·9 (−1·0 to 4·9)	−7·5 (−12·6 to −2·7)†	−0·3 (−3·9 to 3·4)	2·7 (−0·8 to 6·0)	1·4 (−1·7 to 4·3)
Bimanual Fine Motor Function											
	I Without limitation	0·0 (reference)	0·0	0·0	0·0	0·0	0·0	0·0	0·0	0·0	0·0
	II & III Moderate restrictions	−2·2 (−4·2 to −0·2)‡	−1·3 (−3·1 to 0·5)	−1·3 (−3·2 to 0·6)	−1·8 (−3·7 to 0·1)	−2·4 (−4·3 to −0·5)‡	0·1 (−1·8 to 2·0)	−3·5 (−5·9 to −1·1)†	1·0 (−1·0 to 3·0)	0·3 (−1·7 to 2·2)	−0·3 (−2·3 to 1·7)
	IV & V Severe restrictions	−3·3 (−7·5 to 0·8)	−0·2 (−3·6 to 3·3)	0·0 (−3·9 to 4·0)	−1·7 (−5·0 to 1·6)	−3·8 (−7·2 to −0·3)‡	1·0 (−2·2 to 4·1)	−5·6 (−11·3 to −1·4)†	−2·5 (−6·5 to 1·3)	1·7 (−1·5 to 4·8)	0·5 (−3·6 to 2·5)
Seizures											
	No seizures (with or without medication)	0·0 (reference)	0·0	0·0	0·0	0·0	0·0	0·0	0·0	0·0	0·0
	Seizures	−1·6 (−4·8 to 1·7)	−1·0 (−4·1 to 2·1)	−5·3 (−8·2 to −2·3)†	1·2 (−2·2 to 4·6)	0·4 (−2·8 to 3·6)	−1·2 (−4·6 to 2·3)	−1·2 (−5·3 to 2·8)	2·5 (−1·6 to 6·3)	0·4 (−2·9 to 4·0)	−0·7 (−4·5 to 2·8)
Feeding											
	No problems	0·0 (reference)	0·0	0·0	0·0	0·0	0·0	0·0	0·0	0·0	0·0
	Feeds orally with difficulty, or by tube	0·2 (−3·7 to 4·0)	0·7 (−2·5 to 3·9)	−1·1 (−5·0 to 3·0)	−0·5 (−3·8 to 2·8)	−1·1 (−4·9 to 2·5)	0·1 (−3·3 to 3·4)	−5·8 (−11·1 to −1·0)‡	−1·4 (−5·7 to 2·8)	2·9 (−0·4 to 6·2)	0·5 (−1·6 to 4·4)
Intellectual impairment											
	IQ >70	0·0 (reference)	0·0	0·0	0·0	0·0	0·0	0·0	0·0	0·0	0·0
	IQ ≤70	−0·1 (−2·2 to 2·0)	0·3 (−1·6 to 2·2)	−0·7 (−2·8 to 1·5)	1·6 (−0·4 to 3·6)	−1·2 (−3·2 to 0·8)	−0·4 (−2·3 to 1·4)	−4·4 (−7·1 to −1·8)†	0·3 (−1·9 to 2·4)	−1·6 (−3·7 to 0·4)	−2·0 (−4·1 to 0·0)
Communication											
	Normal	0·0 (reference)	0·0	0·0	0·0	0·0	0·0	0·0	0·0	0·0	0·0
	Difficulties	−1·4 (−4·1 to 1·3)	−0·3 (−2·6 to 2·0)	−0·1 (−2·6 to 2·5)	−1·8 (−4·0 to 0·5)	−3·5 (−5·6 to −1·2)†	−1·5 (−3·7 to 0·8)	−4·7 (−7·5 to −2·0)†	−0·5 (−3·0 to 2·1)	−1·0 (−3·5 to 1·4)	−3·4 (−6·1 to −0·9)†
Cerebral palsy type											
	Unilateral spastic	0·0 (reference)	0·0	0·0	0·0	0·0	0·0	0·0	0·0	0·0	0·0
	Bilateral spastic	−0·9 (−3·0 to 1·3)	1·0 (−1·0 to 2·9)	0·5 (−1·5 to 2·6)	0·5 (−1·5 to 2·5)	−0·9 (−2·9 to 1·0)	1·9 (−0·0 to 3·8)	−1·9 (−4·3 to 0·7)	1·1 (−1·1 to 3·2)	0·2 (−1·9 to 2·30	0·7 (−1·3 to 2·7)
	Dyskinetic	0·7 (−3·4 to 4·7)	1·4 (−2·4 to 5·3)	−1·5 (−5·4 to 2·5)	−0·6 (−4·7 to 3·7)	−2·0 (−6·0 to 2·1)	1·2 (−2·3 to 4·7)	−3·1 (−8·2 to 1·5)	−0·8 (−4·9 to 3·4)	2·2 (−1·2 to 5·8)	−1·1 (−5·7 to 3·1)
	Ataxic	−1·5 (−6·6 to 3·9)	1·7 (−2·3 to 6·2)	1·0 (−3·5 to 5·7)	2·9 (−2·4 to 9·1)	−2·9 (−8·0 to 2·8)	−0·3 (−5·2 to 5·2)	−4·5 (−14·2 to 6·2)	−0·3 (−5·8 to 6·2)	1·2 (−5·3 to 7·6)	5·2 (−0·1 to 9·5)
Final models combining significant impairments*											
Gross Motor Function Classification System											
	I Walks and climbs stairs, without limitation	..	..	..	..	0·0 (reference)	..	0·0 (reference)	..	..	..
	II, III, IV, V Limited walking ability or unable to walk	..	..	..	..	−3·4 (−5·2 to −1·6)†	..	−3·5 (−5·8 to −1·1)†	..	..	..
Seizures											
	No seizures (with or without medication)	..	..	0·0 (reference)	..	..	..	..	..	..	..
	Seizures	..	..	−5·3 (−8·2 to −2·3)†	..	..	..	..	..	..	..
Intellectual impairment											
	IQ >70	..	..	..	..	..	..	0·0 (reference)	..	..	..
	IQ ≤70	..	..	..	..	..	..	−3·7 (−6·2 to −1·1)†	..	..	..
*R*^2^, % (95%CI)		..	..	11% (5 to 19)	..	10% (4 to 18)	..	12% (6 to 18)	..	..	..

Data are β coefficient (95% CI) unless otherwise stated.

* Both initial and final models were adjusted for region, age, and sex. β coefficients show the average difference in quality of life between the relevant category and the reference category; β coefficients less than 0 show impaired quality of life. CIs were calculated by bootstrapping. Statistical significance:

† 99% CI excluding zero

‡ 95% CI excluding zero.

After controlling for walking ability, no other impairments were associated with QoL on the Autonomy domain. Likewise, after controlling for walking ability and IQ, no other impairments were associated with QoL on the Social support and peers domain. Hence, the robust associations were as follows: seizures in the previous year were associated with reduced QoL for Moods and emotions; impaired walking ability was associated with reduced Autonomy; impaired walking ability and IQ <70 were associated with reduced Social support and peers (final models, table 5). Typically, these impairments were associated with an average reduction in QoL of between 3 and 5 points (ie, <0·5 SD of QoL in the reference population). For these domains, impairments, age, sex, and region together explained 10–12% of the variation in QoL (final models, table 5). In a post hoc model omitting the adjusting variables, impairment alone explained up to 6% of the variation (data not shown).

More than 95% of 355 participants included in the longitudinal analysis reported at both timepoints on all domains, apart from financial resources (table 6). Changes at group level were small (<3 points), with QoL decreasing in all domains in five domains (p<0·05), increasing in two domains, and not changing significantly (p>0·05) in the remaining three domains. However, individual participants could have large changes in QoL; between 34% and 51% of participants, dependent on domain, showed changes that were more than 10 points (1 SD) in either direction (data not shown).

**Table 6:** KIDSCREEN scores on each domain for young people with cerebral palsy who self-reported in both childhood and adolescence (longitudinal sample, n=355)

	**Number (%)**	**Childhood QoL, mean (SD)**	**Adolescent QoL, mean (SD)**	**Change in QoL between childhood and adolescence**	
				Mean (SD)	p value from paired *t* test
Physical wellbeing	349 (98%)	50·9 (11·7)	49·2 (9·9)	−1·7 (13·1)	0·01
Psychological wellbeing	352 (99%)	51·5 (9·3)	48·6 (9·1)	−2·9 (11·1)	<0·0001
Moods and emotions	347 (98%)	52·0 (10·1)	51·8 (10·0)	−0·2 (12·2)	0·77
Self-perception	347 (98%)	53·4 (10·4)	51·4 (10·0)	−2·1 (12·0)	0·001
Autonomy	353 (99%)	50·1 (10·0)	50·9 (9·7)	0·7 (12·2)	0·26
Relationships with parents	350 (99%)	52·0 (9·3)	51·1 (9·6)	−0·9 (11·4)	0·16
Social support and peers	354 (99%)	48·5 (12·4)	46·3 (12·5)	−2·3 (15·5)	0·006
School life	347 (98%)	55·9 (11·6)	53·1 (10·2)	−2·8 (13·2)	<0·0001
Financial resources	305 (86%)	48·1 (11·3)	50·2 (9·8)	2·1 (14·0)	0·01
Social acceptance	338 (95%)	49·1 (11·0)	50·7 (10·2)	1·6 (13·9)	0·04

QoL=quality of life.

The β coefficients in table 7 show the change in adolescent QoL associated with a change of 1 point in the continuous covariates, but here we give examples of typical changes in QoL associated with a change of 1 SD in these covariates, which might be more clinically relevant. For clarity in table 7 we present β coefficients to one decimal place but the calculations below are based on the values to two decimal places.

**Table 7:** Determinants of self-reported quality of life (QoL) in adolescents with cerebral palsy: regression coefficients of the baseline, pain, Parenting Stress Index (PSI), Strength and Difficulties Questionnaire (SDQ), and combined models (longitudinal sample, n=355)

			**Physical wellbeing**	**Psychological wellbeing**	**Moods and emotions**	**Self-perception**	**Autonomy**	**Relationships with parents**	**Social support and peers**	**School life**	**Financial resources**	**Social acceptance**
**Baseline models**												
Corresponding KIDSCREEN domain in childhood			0·2 (0·1 to 0·3)*	0·3 (0·1 to 0·4)*	0·2 (0·1 to 0·3)*	0·3 (0·2 to 0·4)*	0·2 (0·1 to 0·3)*	0·2 (0·1 to 0·4)*	0·2 (0·1 to 0·3)*	0·2 (0·1 to 0·3)*	0·1 (0·0 to 0·2)	0·1 (0·0 to 0·2)†
*R*^2^, % (95% CI)			14% (11 to 24)	12% (9 to 22)	17% (13 to 30)	16% (11 to 26)	11% (8 to 21)	13% (9 to 23)	14% (10 to 26)	9% (7 to 18)	7% (4 to 17)	16% (12 to 27)
**Pain models**												
Corresponding KIDSCREEN domain in childhood			0·2 (0·0 to 0·2)*	0·2 (0·1 to 0·3)*	0·2 (0·1 to 0·3)*	0·3 (0·2 to 0·4)*	0·2 (0·1 to 0·3)*	0·2 (0·1 to 0·3)*	0·2 (0·1 to 0·3)*	0·2 (0·1 to 0·3)*	0·1 (0·0 to 0·2)	0·1 (0·0 to 0·2)*
Frequency of pain in previous week												
	In childhood											
		None	0·0 (reference)	0·0	0·0	0·0	0·0	0·0	0·0	0·0	0·0	0·0
		Once or twice	−0·7 (−3·1 to 1·7)	−2·6 (−4·7 to −0·5)†	−4·0 (−6·5 to −1·4)*	−1·4 (−3·8 to 1·0)	−1·3 (−3·8 to 1·5)	−2·5 (−4·9 to −0·2)†	−0·9 (−4·1 to 2·4)	−1·8 (−4·4 to 0·9)	0·2 (−2·4 to 2·7)	1·5 (−1·0 to 4·0)
		Frequent	0·4 (−2·2 to 2·9)	−2·7 (−5·0 to −0·5)†	−2·7 (−5·1 to −0·2)†	−1·4 (−4·0 to 1·3)	−1·5 (−4·1 to 0·9)	−2·9 (−5·3 to −0·4)†	−1·9 (−5·3 to 1·7)	−1·2 (−3·7 to 1·2)	−0·7 (−3·3 to 2·0)	−0·2 (−2·9 to 2·3)
	In adolescence											
		None	0·0 (reference)	0·0	0·0	0·0	0·0	0·0	0·0	0·0	0·0	0·0
		Once or twice	−2·1 (−4·5 to 0·3)	−3·4 (−5·7 to −1·1)*	−3·2 (−5·6 to −0·6)†	−3·8 (−6·4 to −1·3)*	−2·2 (−4·7 to 0·4)	−1·9 (−4·4 to 0·5)	−1·8 (−5·2 to 1·3)	−1·8 (−4·4 to 0·7)	−1·4 (−4·1 to 1·2)	−2·6 (−5·3 to −0·2)†
		Frequent	−6·3 (−9·0 to −3·8)*	−5·0 (−7·5 to −2·8)*	−5·4 (−7·7 to −2·9)*	−5·2 (−7·8 to −2·7)*	−3·0 (−5·6 to −0·4)†	−2·6 (−5·1 to 0·0)	−1·8 (−5·1 to 1·5)	−3·6 (−6·4 to −0·9)†	−2·2 (−5·2 to 0·5)	−5·0 (−7·7 to −2·5)*
*R*^2^, % (95% CI)			20% (18 to 32)	19% (15 to 30)	24% (21 to 37)	21% (16 to 32)	13% (11 to 24)	16% (13 to 28)	14% (12 to 28)	12% (9 to 22)	8% (6 to 19)	20% (17 to 32)
**PSI models**												
Corresponding KIDSCREEN domain in childhood			0·2 (0·0 to 0·2)*	0·2 (0·1 to 0·3)*	0·2 (0·1 to 0·3)*	0·3 (0·2 to 0·4)*	0·2 (0·1 to 0·3)*	0·2 (0·1 to 0·3)*	0·2 (0·1 to 0·3)*	0·2 (0·1 to 0·3)*	0·1 (0·0 to 0·2)	0·1 (0·0 to 0·2)*
Total PSI score in childhood			−0·1 (−0·1 to 0·0)†	−0·1 (−0·1 to 0·0)*	0·0 (−0·1 to 0·0)	0·0 (−0·1 to 0·1)	−0·1 (−0·1 to 0·0)*	−0·1 (−0·1 to 0·0)*	−0·1 (−0·2 to 0·0)†	0·0 (−0·1 to 0·1)	0·0 (−0·1 to 0·0)	−0·1 (−0·1 to 0·0)
Change in PSI			−0·1 (−0·2 to 0·0)*	−0·1 (−0·2 to 0·0)*	−0·1 (−0·1 to 0·0)*	0·0 (−0·1 to 0·0)	−0·1 (−0·2 to 0·0)*	−0·1 (−0·1 to 0·0)*	−0·1 (−0·2 to 0·0)†	−0·1 (−0·1 to 0·0)*	−0·1 (−0·1 to 0·0)	−0·1 (−0·1 to 0·0)*
*R*^2^, % (95% CI)			17% (13 to 29)	16% (12 to 25)	18% (15 to 32)	16% (12 to 27)	14% (13 to 25)	16% (13 to 27)	16% (13 to 29)	11% (8 to 21)	8% (5 to 18)	18% (16 to 30)
**SDQ models**												
Corresponding KIDSCREEN domain in childhood			0·2 (0·0 to 0·2)*	0·2 (0·1 to 0·3)*	0·2 (0·1 to 0·3)*	0·3 (0·2 to 0·4)*	0·2 (0·1 to 0·3)*	0·2 (0·1 to 0·4)*	0·2 (0·1 to 0·3)*	0·2 (0·1 to 0·3)*	0·1 (0·0 to 0·2)	0·1 (0·0 to 0·2)†
Total SDQ Score in childhood			−0·3 (−0·4 to −0·1)†	−0·3 (−0·4 to −0·1)	−0·2 (−0·4 to 0·0)†	0·0 (−0·2 to 0·2)	−0·2 (−0·4 to 0·0)†	−0·1 (−0·3 to 0·1)	−0·3 (−0·6 to −0·1)*	−0·2 (−0·4 to 0·0)	−0·2 (−0·4 to 0·0)†	−0·2 (−0·4 to −0·1)†
Change in SDQ			−0·2 (−0·4 to 0·0)	−0·2 (−0·4 to 0·0)†	−0·4 (−0·6 to −0·2)*	−0·2 (−0·4 to 0·0)	−0·2 (−0·5 to 0·0)†	−0·1 (−0·4 to 0·1)	−0·5 (−0·7 to −0·2)*	−0·3 (−0·6 to −0·1)*	−0·2 (−0·5 to 0.0)	−0.4 (−0.6 to −0.2)*
*R*^2^, % (95% CI)			15% (13 to 27)	15% (11 to 25)	21% (17 to 34)	17% (13 to 28)	12% (10 to 23)	13% (11 to 24)	17% (14 to 29)	11% (8 to 21)	9% (6 to 19)	19% (16 to 31)
**Combined models**												
Corresponding KIDSCREEN domain in childhood			0·1 (0·0 to 0·2)†	0·2 (0·1 to 0·3)*	0·2 (0·1 to 0·3)*	0·3 (0·2 to 0·4)*	0·1 (0·0 to 0·2)†	0·2 (0·1 to 0·3)*	0·2 (0·1 to 0·3)*	0·2 (0·1 to 0·3)*	0·1 (−0·1 to 0·2)	0·1 (0·0 to 0·2)†
Frequency of pain in previous week												
	In childhood											
		None	0·0 (reference)	0·0	0·0	0·0	0·0	0·0	0·0	0·0	0·0	0·0
		Once or twice	−0·6 (−3·1 to 1·8)	−2·5 (−4·7 to −0·5)†	−4·3 (−6·8 to −1·7)*	−1·6 (−4·0 to 0·9)	−1·2 (−3·8 to 1·4)	−2·4 (−4·8 to 0·0)†	−0·9 (−3·9 to 2·2)	−1·9 (−4·5 to 0·9)	0·1 (−2·5 to 2·6)	1·4 (−1·2 to 3·9)
		Frequent	0·7 (−1·9 to 3·2)	−2·4 (−4·8 to −0·2)†	−2·5 (−4·9 to −0·2)†	−1·5 (−4·2 to 1·1)	−1·4 (−3·9 to 1·0)	−2·9 (−5·2 to −0·5)†	−1·5 (−4·7 to 2·0)	−1·1 (−3·5 to 1·3)	−0·4 (−3·0 to 2·2)	0·0 (−2·7 to 2·5)
	In adolescence											
		None	0·0 (reference)	0·0	0·0	0·0	0·0	0·0	0·0	0·0	0·0	0·0
		Once or twice	−1·8 (−4·2 to 0·6)	−3·1 (−5·4 to −0·9)*	−2·9 (−5·4 to −0·4)†	−3·8 (−6·3 to −1·3)*	−2·0 (−4·5 to 0·6)	−1·9 (−4·3 to 0·6)	−1·5 (−4·8 to 1·6)	−1·4 (−4·0 to 1·1)	−1·1 (−3·9 to 1·6)	−2·3 (−4·9 to 0·0)*
		Frequent	−5·9 (−8·5 to −3·3)*	−4·5 (−6·9 to −2·3)*	−4·9 (−7·2 to −2·6)*	−5·1 (−7·7 to −2·5)*	−2·4 (−5·0 to 0·3)	−2·1 (−4·6 to 0·5)	−1·0 (−4·3 to 2·2)	−3·2 (−6·1 to −0·3)†	−1·9 (−4·8 to 0·9)	−4·5 (−7·1 to −2·0)*
Total PSI score in childhood			0·0 (−0·1 to 0·0)	0·0 (−0·1 to 0·0)	0·0 (−0·1 to 0·1)	0·0 (−0·1 to 0·1)	−0·1 (−0·1 to 0·0)†	−0·1 (−0·2 to 0·0)	−0·1 (−0·1 to 0·0)	0·0 (0·0 to 0·1)	0·0 (−0·1 to 0·1)	0·0 (−0·1 to 0·0)
Change in PSI			−0·1 (−0·1 to 0·0)†	−0·1 (−0·1 to 0·0)†	0·0 (−0·1 to 0·1)	0·0 (−0·1 to 0·1)	−0·1 (−0·2 to 0·0)†	−0·1 (−0·2 to 0·0)	−0·1 (−0·1 to 0·0)	0·0 (−0·1 to 0·0)	0·0 (−0·1 to 0·0)	0·0 (−0·1 to 0·0)
Total SDQ score in childhood			−0·2 (−0·4 to 0·1)	−0·1 (−0·4 to 0·1)	−0·2 (−0·4 to 0·0)	0·0 (−0·2 to 0·3)	0·0 (−0·3 to 0·2)	0·1 (−0·1 to 0·3)	−0·2 (−0·5 to 0·1)	−0·2 (−0·5 to 0·1)	−0·2 (−0·4 to 0·1)	−0·1 (−0·4 to 0·1)
Change in SDQ			0·0 (−0·3 to 0·2)	−0·1 (−0·3 to 0·2)	−0·4 (−0·6 to −0·2)*	−0·2 (−0·5 to 0·1)	0·0 (−0·3 to 0·2)	0·1 (−0·2 to 0·3)	−0·3 (−0·7 to 0·0)†	−0·2 (−0·5 to 0·0)	−0·1 (−0·4 to 0·1)	−0·3 (−0·6 to 0·0)†
*R*^2^, % (95% CI)			22% (20 to 35)	22% (18 to 34)	28% (25 to 41)	22% (18 to 34)	16% (15 to 29)	19% (16 to 32)	18% (16 to 32)	14% (12 to 26)	9% (8 to 21)	22% (21 to 36)

Data are β coefficient (95% CI) unless otherwise stated. β coefficients for continuous covariates (QoL in childhood, SDQ, and PSI scores, changes in SDQ and PSI scores) show the average change in QoL in adolescence associated with a change of 1 point in the covariate. β coefficients for categorical covariates (pain) show the estimated average difference in QoL between the relevant category and the reference category. All regression models were adjusted for region, age, sex, and impairments that were significant in cross-sectional analyses. Changes in PSI and SDQ were calculated as adolescent scores minus childhood scores. CIs were calculated by bootstrapping. Statistical significance:

* 99% CI excluding zero

† 95% CI excluding zero.

Childhood QoL was a significant predictor of adolescent QoL in all domains apart from Financial resources (baseline models, table 7). The association was strongest for Self-perception, in which an increase of 10 points (1 SD) in the QoL score in childhood was associated with an increase in adolescent QoL of 2·8 points (95% CI 1·7–4·0).

Adolescents who reported pain in childhood or in adolescence had lower QoL than did other adolescents in all domains apart from Social support and peers and Financial resources (pain models, table 7); this difference was strongest for the Physical wellbeing domain, where the average QoL of adolescents who reported pain more than twice a week was 6·3 points (95% CI 3·8–9·0) lower than that of those who reported no pain. Pain in childhood was an independent predictor of lower QoL in adolescence in three domains: Psychological wellbeing, Moods and emotions, and Relationships with parents.

Adolescents in families with high parenting stress scores in their childhood had significantly lower QoL than other adolescents in five domains, although the effects were small (PSI models, table 7). For example, for the Autonomy domain an increase of 15 points (1 SD) in the PSI score in childhood was associated with a decrease in adolescent QoL of 1·3 points (95% CI 0·5–2·1). Worsening of parenting stress between childhood and adolescence predicted a small but significant decrease in adolescent QoL in all domains apart from Self-perception and Financial resources.

Adolescents who had psychological difficulties in childhood had lower QoL than did other adolescents in all domains apart from Self-perception and Relationships with parents, although the effects were small (SDQ models, table 7); for example, for the Social support and peers domain an increase of 7 points (1 SD) in the SDQ score in childhood was associated with a decrease in adolescent QoL of 2·3 points (95% CI 0·5–4·2). Worsening of psychological difficulties between childhood and adolescence predicted similar reductions in adolescent QoL for six domains (95% CI excluding zero).

In the models combining the postulated predictors of QoL, pain, especially in adolescence, remained a significant predictor of adolescent QoL in all domains apart from Autonomy, Social support and peers, and Financial resources (combined models, table 7). After controlling for pain, PSI was significant on only one domain, Autonomy, and SDQ did not reach significance on any domain. Change in PSI was significant on the domains of Physical wellbeing, Psychological wellbeing, and Autonomy; change in SDQ on the domains of Moods and emotions, Social support and peers, and Social acceptance. Depending on domain, the models explained between 9% and 28% (*R*^2^) of the variation in adolescent QoL.

Sensitivity analysis, imputing missing KIDSCREEN scores for participants who self-reported on only one occasion, gave β coefficients and *R*^2^ that were generally smaller than those from the primary analysis (data not shown). Complete case analyses gave much the same results to those in table 7, although with slightly increased β coefficients (data not shown).

## Discussion

We report the QoL of a large, representative sample of adolescents with cerebral palsy who could self-report. Our results are encouraging. On only one domain, Social support and peers, was the average QoL of these adolescents significantly lower than that of their able-bodied peers—by 2·7 points or about 0·25 SD. Furthermore, severe impairment was associated with low QoL on only three domains: active seizures with Moods and emotions, and a number of correlated impairments with Autonomy and Social support and peers. Average differences between the least and most able groups were generally less than 0·5 SD. Our study excluded individuals who could not self-report due to severe learning difficulties; these young people have a higher prevalence than self-reporting adolescents of severe impairments and thus the pattern of proxy-reported QoL for this group might be different. We will analyse proxy measures of their QoL in a future report.

To our knowledge, our longitudinal study is the first to track QoL of young people with cerebral palsy from childhood to adolescence (panel). We noted that adolescent QoL had strong associations with pain but only slight associations with child QoL, parenting stress, and child psychological problems. Compared with adolescents without pain, the QoL of those with frequent pain was up to 6 points lower, dependent on domain. If QoL, PSI, and SDQ in childhood were 1 SD worse (equating to a 10 point decrease in QoL, a 15 point decrease in PSI, and a 7 point decrease in SDQ scores), the predicted average adolescent QoL would be lower by 1–3 points for QoL, 0–1·3 points for PSI, and 0–2·3 points for SDQ, dependent on domain assessed. Worsening of SDQ between childhood and adolescence by 7 points predicted a 1–3 point reduction in adolescent QoL.

Panel **Research in context**

**Systematic review**

We searched PubMed and Web of Science with the search terms “cerebral” AND “palsy” AND “quality” AND “life”, to identify reports published since Jan 1, 1990. We also undertook hand searches of references in identified reports. Conflicting results have been reported from cross-sectional studies of adolescents with cerebral palsy about variation of QoL with impairment^6^, ^8^, ^9^ and comparison with the general population.^5^, ^10^ These inconsistencies are probably because of small sample sizes;^5^, ^6^, ^8^, ^9^, ^10^ combination of self-reports and proxy reports,^8^ inclusion of different age groups; and inappropriate choice of methods to capture QoL.^6^, ^9^, ^10^, ^29^ We identified very few longitudinal studies, and all were short (1–3 years^30^, ^31^, ^32^, ^33^), had small samples (<200 individuals), and measured function rather than subjective wellbeing (eg, Health Utilities Index,^30^ Child Health Questionnaire,^31^, ^32^ or TACQOL^33^). As we noted, the authors of these reports noted stability of QoL at the group level. Only one study examined individual variation^30^ finding substantial differences between individuals in how their QoL changed over time, especially in relation to emotion and pain. In the only longitudinal study^33^ that examined predictors of adolescent QoL, mental health problems predicted lower social functioning and mood, consistent with our findings.

**Interpretation**

We believe our study provides the most robust evidence about how young people with cerebral palsy feel about life because of its large sample size, random selection of the sample from population-based registers, capture of a view of QoL related to subjective wellbeing reported by the child or young person, rather than a health-related view that captures function and perceived effect of cerebral palsy, and use of a validated European questionnaire whose psychometric properties are similar in children with cerebral palsy and the general population.^34^ Individual and societal attitudes should be affected by the similarity of the QoL of adolescents with and without cerebral palsy. Only the quality of peer relationships is on average lower in the adolescents with cerebral palsy than adolescents in the general population, and therefore such adolescents need particular help to maintain and develop peer relationships. Interventions in childhood to alleviate psychological difficulties, parenting stress, and especially pain, are justified for their intrinsic value and for their long-term effect on adolescent QoL.

Similar to their counterparts in the general population, some adolescents with cerebral palsy have low QoL and parents, clinicians, and carers need to understand how to help such individuals and, if possible, prevent the emergence of low QoL. Our longitudinal study provided some suggestions. The modifiable factors—pain, parenting stress, and psychological problems—are more prevalent in children with cerebral palsy than in the general population,^35^, ^36^, ^37^, ^38^ so whether their associations with adolescent QoL are causal needs to be assessed. Intervention studies, ideally randomised clinical trials, would provide the best evidence about causality. However, a recent systematic review of clinical trials^39^ to improve QoL in children and adolescents with cerebral palsy did not find any trials that addressed pain, psychological problems, and parenting stress. We noted the magnitude of the association between adolescent QoL and adolescent pain was large, providing support for a causal interpretation.^40^ However, the magnitude of the associations with childhood QoL, PSI, and SDQ were slight. The longitudinal nature of our study provides support for a causal interpretation of these associations, as does the plausibility of the associations and their consistency across most domains.^40^ Therefore, clinicians should intervene early in childhood to ameliorate extremes of pain, psychological problems, and parenting stress, for which effective interventions are available.^41^, ^42^, ^43^, ^44^, ^45^ Such interventions would be valuable not only for their intrinsic worth but also for their potential to affect both adolescent QoL and contemporaneous QoL.^3^, ^12^ Pain in particular has a pervasive association with QoL in both childhood and adolescence and needs to be asked about and dealt with in clinical consultations.^46^, ^47^

We were concerned to note a reduction in QoL on the domain of Social support and peers in our cohort, especially in more severely impaired adolescents, because this finding was not apparent in the same children in SPARCLE1.^3^ Therefore, attention should be directed to helping children with cerebral palsy, especially those who are more severely impaired, to maintain friendships with peers, to develop new friendships as they move into adolescence, and to participate fully in society. The association between seizures and low moods and emotions emphasises the importance of striving to control seizures.

It might appear inconsistent that lower adolescent QoL is associated with factors such as pain or psychological problems, which are more prevalent in young people with cerebral palsy,^35^, ^36^, ^37^, ^38^ whereas our cross-sectional comparison showed that adolescents with cerebral palsy had higher QoL on five domains than did adolescents in the general population. This finding could be an artifact due to systematic bias because the adolescents with cerebral palsy and their able-bodied peers were selected in different ways. Alternatively, QoL could be determined by different factors in adolescents with and without cerebral palsy. Nonetheless, we conclude that the QoL of adolescents with cerebral palsy is not lower than that of adolescents in the general population (apart from in the domain of Social support and peers). Although we reported that children with cerebral palsy had similar QoL to children in the general population,^3^ we did not know if adolescence (which is a challenging time) might lead to unhappiness if the individual also had cerebral palsy; so this finding is encouraging. However, an equally important outcome, although not the subject of this report, is participation or involvement in life situations (ranging from leisure pursuits to education and social roles),^48^ which is strikingly reduced in those with cerebral palsy, both in childhood^49^, ^50^ and adolescence.^51^, ^52^, ^53^, ^54^

Our study provides some of the most reliable evidence on how adolescents with cerebral palsy feel about life (panel).^34^ The main concern about the validity of our study is differential non-response. We tried to minimise its possible effects in three ways. First, we used multiple imputation to impute missing values for all individuals who self-reported QoL in SPARCLE2; this technique can help to reduce bias and increase precision when data are missing.^55^ Second, because non-response by families targeted for recruitment to SPARCLE1 was 37%,^14^ we adjusted for region and walking ability, which were predictors of non-response.^56^ Third, because 25% of children who self-reported QoL in SPARCLE1 dropped out in SPARCLE2, and non-response in the SPARCLE2 supplementary sample was high (76%),^16^ we did a sensitivity analysis that included all children who self-reported in either SPARCLE1 or SPARCLE2, imputing missing KIDSCREEN scores, and excluded the supplementary sample. The sensitivity analysis yielded similar results to the primary analysis. Nevertheless, findings for the Financial resources domain should be interpreted with caution because of the extent of missing data and the ceiling effect in this domain.

We also tried to ensure that any deviations of KIDSCREEN scores from normality did not affect our analyses, first by use of non-parametric methods for the comparison between adolescents with cerebral palsy and adolescents in the general population and, second, by use of bootstrapping (a technique that does not assume normality) to estimate the CIs in our regression models.

To restrict the possibility that some results might appear significant by chance, we examined 99% and 95% CIs, and in our interpretation we took into account the findings of sensitivity analyses that generated fewer significant results.

The differences between individuals with cerebral palsy and those in the general population could be due to factors other than the presence or absence of cerebral palsy. Adolescents with cerebral palsy were selected from specific regions within each country, whereas the adolescents in the general population were selected from the country as a whole. The two samples completed the questionnaires at different times, individuals with cerebral palsy in 2009–10 and those in the general population in 2003.^18^ The two samples were selected in different ways, individuals with cerebral palsy from population-based registers and those in the general population from representative schools in Sweden, the UK, and Denmark, and through computer-assisted telephone interviews in France and Germany.^18^

Trials are needed to address factors such as pain, psychological problems, and parenting stress in which QoL is a primary or secondary outcome and follow-up is for years rather than months. Qualitative research is needed to understand better why peer relationships seem to worsen for many young people with cerebral palsy between childhood and adolescence. Continuing the SPARCLE study into young adulthood would reveal if this worsening persists. Finally, new longitudinal studies are needed to investigate other factors that might explain the variance in QoL such as personality traits, participation, or parenting styles.

Individual and societal attitudes should be affected by the similarity we identified in the QoL of adolescents with and without cerebral palsy, although young people with cerebral palsy need particular help with maintaining and developing peer relationships. Although the rights of people with disabilities to participate in society are recognised and being implemented in many countries, adolescents with disabilities might still be regarded as having unhappy, unfulfilled lives. Findings from qualitative studies,^57^, ^58^, ^59^ which challenge such a view, are now supported by our large epidemiological study. For children with below average QoL, early interventions to ameliorate high levels of psychological problems, parenting stress, and especially child pain, will probably have long-term benefits across many domains of the young person's life.
