# Supplementary material for: Self-reported quality of life of adolescents with cerebral palsy: a cross-sectional and longitudinal analysis
Source: Lancet. 2015 Feb 21;385(9969):705–16. doi: 10.1016/S0140-6736(14)61229-0 (PMC4606972; doi:10.1016/S0140-6736(14)61229-0)

THE LANCET

Supplementary appendix

This appendix formed part of the original submission and has been peer reviewed. We post it as supplied by the authors.

Supplement to: Colver A, Rapp M, Eisemann N, et al. Self-reported quality of life of adolescents with cerebral palsy: a cross-sectional and longitudinal analysis. *Lancet* 2014; published online Oct 7. http://dx.doi.org/10.1016/S0140-6736(14)61229-0.

Appendix: Full statistical methods

The provenance of the cross-sectional and longitudinal samples is summarised in the Figure. Drop-out between SPARCLE1 and SPARCLE2 varied significantly between regions; it also varied with parental educational qualifications, parental stress, family structure and child pain, those with lower educational qualifications, higher levels of stress, more frequent pain and the unmarried being more likely to drop out.[1] However, we found no significant difference between the self-reported QoL in childhood of those who did and did not drop out between SPARCLE1 and SPARCLE2.
We first examined the psychometric properties (floor and ceiling effects, Cronbach's alpha) of the self-reported KIDSCREEN scores in adolescents with CP, using the cross-sectional sample (n=431). We then assessed the change in QoL between childhood and adolescence using paired t-tests on the longitudinal sample (n = 355). Before further analysis, we generated ten *imputed datasets* using multiple imputation with chained equations[2] for all young people who self-reported QoL in SPARCLE2: we imputed missing values of impairment, QoL, PSI and SDQ scores, and pain from observed values of age, gender, region, walking ability, family structure, parental educational qualifications, PSI and SDQ in SPARCLE1, using polytomous regression for categorical variables and predictive mean matching for interval scaled variables. In the analyses described below, we obtained point estimates using Rubin's rules; 95% and 99% confidence intervals (CI) were estimated by bootstrapping methods[3] with 100 replications per domain for each of the ten imputed datasets. The criterion for statistical significance was that the 95%CIs did not include zero; but in our interpretation we took into account both 99%CI and findings of sensitivity analyses which generated fewer apparently significant results.

Cross-sectional analysis: comparison of adolescents with CP with the general population
We compared the QoL of adolescents with CP in the cross-sectional sample (n=431) with the QoL of adolescents in the general population. We excluded from the comparison: those with CP in Italy (n=17) as no data on the general population were available for Italy; those with CP in Sweden who were aged 16 or 17 (n=13) and those with CP in Denmark aged 12 (n=2), as no data on the general population were available for adolescents of those ages in those countries. These exclusions reduced the sample of adolescents with CP to 399.
The distribution of age, gender and country was significantly different among adolescents with CP and those in the general population. We therefore controlled for these factors by selecting two controls from the general population for each adolescent with CP, matching on age, gender and country. We then estimated the mean difference of the QoL of adolescents with CP and their matched controls.

Cross-sectional analysis: variation of QoL with impairment in adolescents with CP
We analysed the relationship between impairment and QoL in adolescence using linear regression, adjusted for region, which had partly determined the sampling strategy, and for gender and age, which are known correlates of QoL of adolescents.[4] We treated these variables as fixed effects, age as a continuous variable and impairments as categorical variables. We considered each impairment in turn (initial models). We checked whether significant impairments (i.e. those with 95%CI excluding zero) remained significant after allowing for walking ability, which had partly determined the sampling strategy.[5] If more than one impairment was significant, we combined significant impairments in a final model using a forwards stepwise procedure that preferentially included walking ability and then IQ. For the final model, we noted the R^2 statistic as an estimate of the percentage of variance explained by the combination of impairment and adjusting variables.

Longitudinal analysis: childhood and adolescent predictors of QoL of adolescents with CP
We used linear regression on the longitudinal sample (n=355) to relate QoL in adolescence (outcome variable) to QoL in childhood (independent variable). As before, we adjusted the regression for region, gender and age and additionally adjusted for GMFCS and for those impairments found to be significant in the primary cross-sectional analysis. Following estimation of this baseline model, we investigated the influences on QoL in adolescence of pain, PSI and SDQ in separate models. In addition to the factors considered in the baseline models, the pain model included pain in both childhood and adolescence; the PSI and SDQ models included the respective scores in childhood and their changes between childhood and adolescence. Finally, we conducted a combined regression model in which all these factors (pain, SDQ and PSI) were included.
We treated pain as a categorical variable. Although the SDQ and PSI scores are presented as categorical data in Table 1, we treated these scores (and their changes) as continuous variables in the analysis; regression coefficients indicate the change in adolescent QoL score for a change of one point in SDQ or PSI.

Sensitivity analyses
We performed sensitivity analyses for both cross-sectional and longitudinal analyses. These analyses were performed on all 534 SPARCLE1 participants who self-reported their QoL in either SPARCLE1 or

SPARCLE2, including the 123 (25%) self-reporting children who dropped out between SPARCLE1 and SPARCLE2 and the 34 who dropped out between SPARCLE1 and SPARCLE2 and those who self-reported in SPARCLE2 but not in SPARCLE1 (see Figure). We imputed missing data as described above, followed by the methods described for the primary analysis.

We used the statistical packages Stata12[6] and R[7] for analysis.

References

1. Dickinson HO, Rapp M, Arnaud C, et al. Predictors of drop-out in a multi-centre longitudinal study of participation and quality of life of children with cerebral palsy. *BMC Research Notes* 2012; **5**: 300.

2. van Buuren S. Multiple imputation of discrete and continuous data by fully conditional specification. *Stat Methods Med Res* 2007; **16**: 219-42.

3. Stine R. An introduction to bootstrap methods: Examples and ideas. In: Fox J, Long JS, eds. Modern Methods of Data Analysis. Newbury Park, CA: Sage, 1990:353-73.

4. Bisegger C, Cloetta B, von Rueden U, Abel T, Ravens-Sieberer U. Health-related quality of life: gender differences in childhood and adolescence. *Soz Praventivmed* 2005; **50**: 281-91.

5. Dickinson H, Parkinson K, McManus V, et al. Assessment of data quality in a multi-centre cross-sectional study of participation and quality of life of children with cerebral palsy. *BMC Public Health* 2006; **6**: 273.

6. Stata Statistical Software: Release 12 [program]. College Station, TX: StataCorp LP, 2011.

7. R: A language and environment for statistical computing [program]. 3.0.0 version. Vienna: R Foundation for Statistical Computing, 2013.

Figure. Study Profile

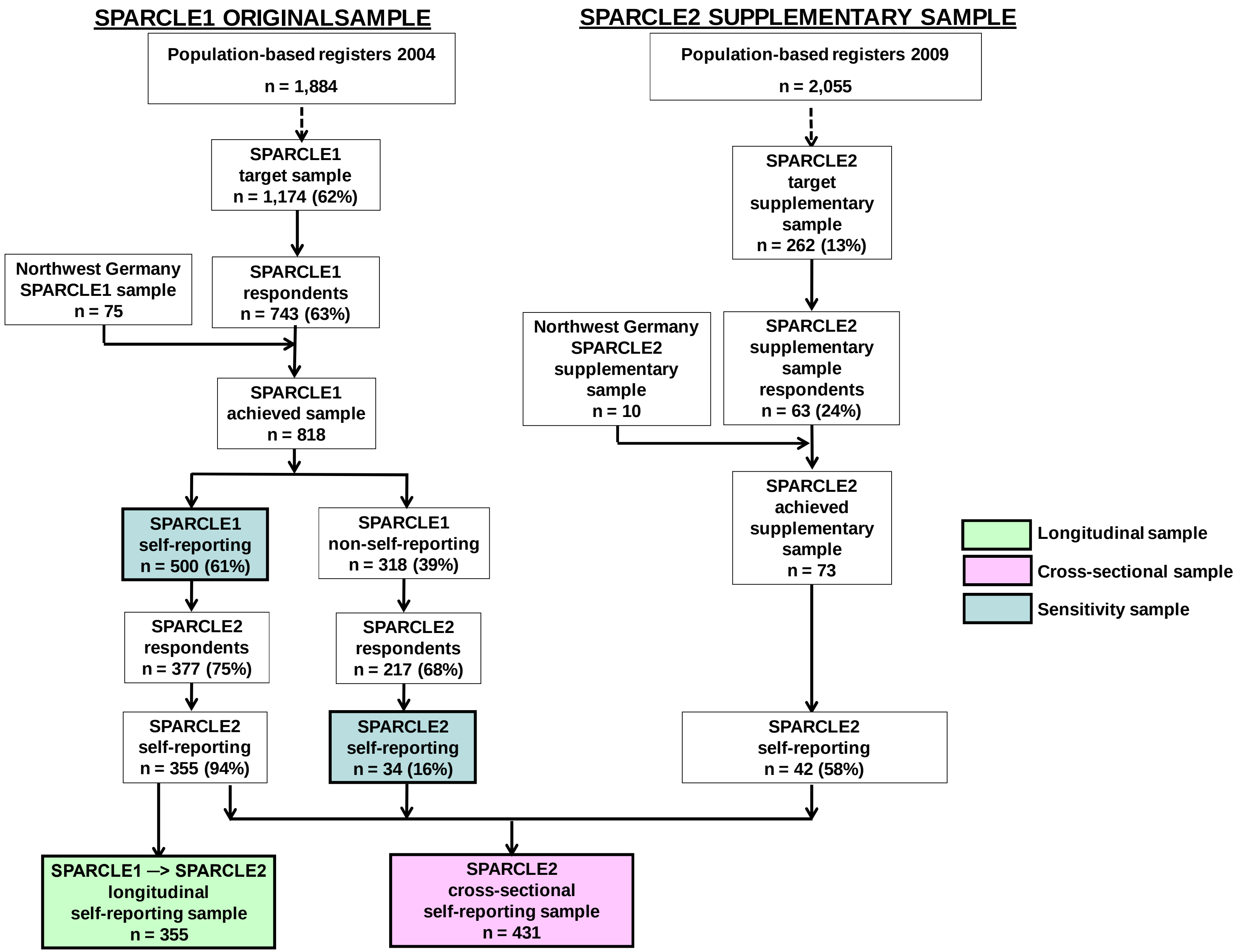

Supplement: Supplementary appendix [file mmc1.pdf]
